# SerpinE2 promotes M2 polarization in macrophage to accelerate colorectal cancer progression

**DOI:** 10.3389/fonc.2025.1585935

**Published:** 2025-05-20

**Authors:** Hui-Long Liu, XiaoSheng Gao, WeiFeng Yang, Wang-Lin Li

**Affiliations:** Department of Colorectal Surgery, the Second Affiliated Hospital, School of Medicine, South China University of Technology, Guangzhou, Guangdong, China

**Keywords:** colon cancer, SerpinE2, tumor-associated macrophages, immune infiltration, M2 polarization

## Abstract

**Background:**

Cell-cell crosstalk in the tumor microenvironment (TME) is crucial for cancer development and strongly correlates with clinical outcomes. Interpatient variability in tumor microenvironment composition and function poses ongoing challenges for personalized therapy selection, remaining a significant clinical problem. Serpin family E member 2 (SERPINE2) released from the tumor microenvironment exhibits significant regulatory functions in cancer progression but the role of SERPINE2 in the tumor microenvironment remains unclear. In this study, we want to investigate the potential mechanism of SERPINE2 in tumor microenvironment of colon cancer.

**Methods:**

Bioinformatics analysis was used for exploring the mRNA expression level of SERPINE family in Pan-cancer, the prognostic significance of SERPINE family overexpression in four cancer types, the clinical relevance of SERPINE2 and the potential function of SERPINE2 in colorectal cancer. We conducted qRT-PCR, Western blot and ELISA to investigate the expression of SERPINE2. Additionally, Tissue chips, Transwell assays, Cell counting kit-8 assay, and co-culture system were used to evaluate the relationship between SERPINE2 and polarization of tumor-associated macrophages.

**Results:**

Based on public database screening, the SERPINE family genes were significantly upregulated in various cancers, and high expression of SERPINE family genes in colorectal cancer was closely associated with poor prognosis. Compared to other family members, SERPINE2 showed a high expression level and was closely related to clinical malignant progression of colon cancer patients. co-expression network analysis, KEGG and GO analysis revealed that SERPINE2 expression correlates with tumor immunoregulation, division and proliferation. Immune infiltration analysis indicated a significant positive correlation between SERPINE2 and M2 macrophage infiltration, and tissue chip confirmed the correlation between SERPINE2 expression in colon cancer tissues and macrophage infiltration. Cell co-culture experiments further demonstrated that SERPINE2 secreted by colon cancer cells can induce polarization of M2 macrophages. Next, the recombinant protein SERPINE2 was observed to stimulate macrophage polarization. We found macrophages induced by SERPINE2 in co-culture with cancer cells accelerated cancer cell proliferation and migration.

**Conclusion:**

Our study demonstrates that tumor-secreted SERPINE2 mediates a positive feedback loop between tumor cells and M2 macrophages to accelerate cancer progression, suggesting SERPINE2 may be as a promising therapeutic target for colon cancer treatment.

## Introduction

Colorectal cancer (CRC) is a prevalent malignant tumor of the digestive tract in clinical practice. In 2020, there were approximately 1.93 million new cases of CRC worldwide, resulting in 940, 000 deaths. This makes it the third most common cancer globally and the second leading cause of cancer-related death worldwide (1, 2). Due to the continuous optimization and adjustment of treatment regimens, A comprehensive strategy combining surgery, radiotherapy, chemotherapy, targeted therapy, and immunotherapy has been developed. Targeted therapy has changed the concept of cancer treatment and colorectal cancer can be improved in clinical efficacy through targeted proteins or genes (3). Currently available targeted drugs are still limited. Therefore, it is urgently needed to discover more new targets in order to optimize existing therapies.

Cancer cells exist within a highly complex tumor microenvironment (TME), consisting of stromal cells, endothelial cells, and immune cells and TME plays a crucial role in the initiation and progression of tumors (4). M0 macrophages, derived from monocytes and recognized as a major innate immune cell type, can be polarized into either classically activated (M1) or alternatively activated (M2) macrophages, collectively referred to as tumor-associated macrophages (TAMs) (5). Tumor-associated macrophages, characterized by their remarkable phenotypic plasticity, represent the predominant immune cell population within the tumor microenvironment (6). TAMs exhibit responses to a variety of microenvironmental stimulus and promote tumor cell proliferation, invasion, and metastasis (7).For instance, when macrophages are cultured in the conditioned medium of colorectal cancer cells, they display a combination of M1/M2 phenotypes and control the EMT program to promote tumor cell migration and invasion (8). The interaction between TAMs and cancer cells is highly intricate. Therefore, the fundamental mechanisms of crosstalk between TAMs and cancer cells may aid in developing efficacious therapeutic strategies to improve the prognosis of patients with colorectal cancer (9).

As a member of the SERPINE superfamily, it possesses a structure consisting of 3β-sheets (A, B, and C) and 9 α-helices (hA to hI) (10). The SERPINE family consists of three members: SERPINE1 (also known as PAI-1), SERPINE2 (also known as protease nexin 1, PN-1) and SERPINE3. It has been documented that the genes of the SERPINE family play crucial roles in the regulation of various cancers and are closely associated with immune infiltration (11).

SERPINE2 is a potent inhibitor of serine proteases, which regulates the activity of plasminogen activators and thrombin, and is involved in numerous biological processes (12). The incidence of various cancers is closely associated with SERPINE2 expression, making it crucial to explore its regulatory mechanism *in vivo*. Research has demonstrated that SERPINE2 promotes extracellular matrix production and local invasion of pancreatic tumors *in vivo* (13). Furthermore, SERPINE2 modulates post-transcriptional regulation via direct physical interactions with effector proteins. Recent studies indicate that SERPINE2/PN-1 plays a role in regulating the DNA damage response and radio resistance in lung cancer through the activation of ATM (14). Due to its complex regulatory mechanisms, elucidating SERPINE2 and its potential regulatory mechanisms will help understand the pathogenesis of colon cancer.

In this study, we mainly investigated the mRNA expression levels of SERPINE family genes in human cancer samples and human normal tissues in the Cancer Genome Atlas (TCGA) database. Then, the Kaplan-Meier Plotter database was used for comprehensively studying the prognostic value of SERPINEs in significantly upregulated cancers. Furthermore, we analyzed SERPINEs at the mRNA and protein level in colorectal cancer and clinical relevance of SERPINE2 in Colorectal Cancer Patients. We predicted potential pathways for SERPINE2 involvement in tumorigenesis through co-expression network analysis and enrichment analysis. As an extracellular secretory factor from colon cancer cells, we analyzed its association with immune infiltrating cells. We discovered an association between SERPINE2 and macrophage infiltration, as well as a interaction between SERPINE2 and the tumor-associated macrophages. In conclusion, our study evaluated the expression levels and prognostic value of SERPINE2 in colorectal cancer, revealing its relationship with the immune tumor microenvironment and interaction with tumor-associated macrophage. This offers new perspectives into targeting SERPINE2 as a potential therapeutic target for colorectal cancer.

## Materials and methods

### Gene expression analysis

We downloaded the gene expression RNAseq (FPKM format) of 33 cancers from TCGA database using UCSC Xena (https://xena.ucsc.edu/) (15). The mRNA expression profile with normal tissue was extracted from Genotype-Tissue Expression(GTEx) database (https://gtexportal.org/home/datasets) to supply normal tissue RNA-seq transcriptome data lacking in TCGA. Number of patients in pan-cancer tissues, the abbreviations and full names of the cancers involved in the study are presented in **Supplementary Table S1**. The perl software was used to extract and integrate the SERPINE family genes expression level. The “Wilcox. test” was also employed to assess the differential SERPINE family gene expression in 33 cancer types. The pictures of the analyses results were drawn with the R-package “ggplot.” Besides, heatmap were designed using the and “pheatmap” R-packages.

### UALCAN

By obtaining data from TCGA database, UALCAN can be used not only to assess the expression of protein-coding genes, but also to conduct in-depth analysis of clinical data in 33 cancers (16). In this study, differences in SERPINEs mRNA expressions and protein expressions between normal and tumor tissues were obtained through “Expression Analysis” module and Colon Adenocarcinoma(COAD) dataset, and t test was used for analysis, with P < 0.05 as the significance threshold.

### GEPIA

GEPIA is an analysis tool based on TCGA and GTEx data, which contains RNA sequence expression data of 9736 tumors and 8587 normal tissue samples. (17) In this study, GEPIA single gene analysis was used to analyze both the difference of SERPINE2 mRNA expression and pathological stage in COAD dataset. We also analyzed the correlation of CD68 and SERPINE2 mRNA expression. T test analysis was used, with P < 0.05 as the significant threshold, and Kaplan–Meier curve was used for prognosis analysis.

### Kaplan-Meier plotter

The KM plotter (http://kmplot.com) database evaluates survival of different genes in 21 cancer types including breast (n=6234), ovarian (n=2190), lung (n=3452) and gastric (n=1440) cancer. (18) Prognostic values including OS (overall survival) of SERPINEs were evaluated in Colon Adenocarcinoma(COAD)/Head and Neck Squamous Cell Carcinoma(HNSC)/Kidney Renal Papillary Cell Carcinoma(KIRC)/Rectum Adenocarcinoma(READ). Twelve different probes were used to evaluate SERPINE2 related OS in the four cancers. Hazard ratios (HRs) with 95% confidence intervals and logrank P-value were determined.

### cBioportal

cBioportal is a platform for exploring, visualizing, and analyzing multi-dimensional cancer genomic data. cBioportal contains over 200 cancer genomics studies from the TCGA database. (19) In this study, a co-expression gene dataset of the SERPINE2 were obtained from cBiopartal based on TCGA database. A total of 348 COAD specimens were analyzed.

### DAVID6.8

DAVID6.8 provides a method for elucidating the biological functions of the submitted genes. (20) In this study, GO enrichment analysis and KEGG pathway enrichment analysis of the coexpression network of SERPINE2 were isolated from DAVID6.8, including Biological Process(BP), Cellular Component(CC), Molecular Function(MF).

### TIMER

TIMER provides a systematic evaluation of different immune cell infiltrates and their clinical effects. (21) In this study, the gene module was used to evaluate the correlation between the SERPINE2 levels and macrophage subtypes infiltration. Besides, analysis of immune cell infiltration was conducted with the Sangerbox platform, a free online platform for data analysis which date was obtained from TIMER in COAD(N=282)(http://www.sangerbox.com/tool).

### Tissue microarray and immunohistochemistry

Two tissue microarrays (TMA) containing 48 cases of tumor tissues and 48 non-tumor tissues were obtained from Shanghai Zhuoli Biotechnology Co, Ltd. (Zhuoli Biotechnology Co, Shanghai, China). All tissue samples were fixed in formalin and embedded in paraffin for future use in IHC.

Immunohistochemistry (IHC) was performed according to the manufacturer’s protocol. After dewaxing with xylene, the samples were rehydrated with ethanol. The tissue samples were incubated with 3% H2O2 for 5 min and incubated with sodium citrate buffer (pH 6.0) for 20 min. Finally, the sections were incubated with primary antibodies against SERPINE2 and CD68 overnight at 40°C, followed by incubation with the secondary antibodies. Two independent pathologists who were blinded assessed the positivity and intensity of the tissue sections. The expression levels were assessed based on the staining intensity (0 for no staining, 1 for weak staining, 2 for moderate staining, and 3 for strong staining) and the positive cell ratio (0 for <10%, 1 for 10 to <50%, and 2 for ≥50% cell). The histochemistry score (H-SCORE) was calculated as follows: H-SCORE = (percentage of cells with weak staining × 1) + (percentage of cells with medium staining × 2) + (percentage of cells with strong staining × 3).The two scores were combined with the following formula: IHC score = positive rate score × intensity score.

### Cell lines and cell culture

The human colon cancer cell lines (HCT-8, HCT-15), a normal human colon mucosal epithelial cell line NCM460 and an acute myeloid leukemia human monocyte (THP-1) were purchased from the American Type Culture Collection (ATCC, USA). Roswell Park Memorial Institute (RPMI-1640, Gibco) supplemented with 10% fetal bovine serum (Gibco) was used to maintain all the cell lines under standard conditions (5% CO2, 37°C).

### THP-1 differentiation and coculture system

To be differentiated into adherent macrophages, THP-1 cells (5 × 10⁵/well) were seeded into 6-well plates in the presence of 100 ng/ml of phorbol 12-myristate 13-acetate (PMA, Sigma) for 24 h. The differentiated macrophages were named as M0 macrophages. To establish a co-culture system, we collected conditioned medium from Normal intestinal cells NCM460 and transfected HCT-8 cells with SERPINE2 siRNA or NC. At 48 h post-transfection, we used the culture supernatant to incubate the M0 macrophages. After 48 h of co-culture, the macrophages were harvested for analysis. Besides, in order to explore the effect of SERPINE2 on macrophage polarization, M0 macrophages were treated with recombinant SERPINE2 (0.4 ng/mL, TargetMol) during 24 hours and then harvested for subsequent experiment.

### Cell counting kit-8 assay

The colorectal cancer cells HCT-8 were seeded into a 96-well plate, treated with the pretreated conditioned medium. The cells were incubated with CCK8 reagent (DingGuo Bio) at 37°C for 2 h and absorbance at 450 nm were measured using a microplate reader (BioTek).

### Transwell assay

Cellular motility and invasive abilities were determined using Transwell (8.0 μm polycarbonate Membrane, Corning Life Sciences, Bedford, MA, USA) and Matrigel invasion (BD Biosciences, San Jose, CA, USA), respectively. For the cell invasion analysis, transwell chambers were placed on 24-well plates; the upper chamber coated with Matrigel was seeded with 2 × 10⁵ cells and cultured with 200 µl serum-free medium, and the lower chamber was added with 600 µl DMEM medium containing 10% FBS. After 24 h of culture, the chambers were removed and fixed with 4% paraformaldehyde at room temperature for 20 min, and then stained with crystal violet at room temperature for 15 min. The cells in the upper chamber were gently wiped off with a cotton swab. Five fields were randomly selected under a microscope and photographed (200×), Image J software was used for cell counting. The migration experiments were performed in the same manner, but the upper chamber was without the Matrigel.

### ELISA

ELISA was conducted according to the instructions. Concentrations of SERPINE2 (human) in the culture supernatant of treated cells were measured with the use of a commercially available kit (CUSABIO).Seed HCT-8 cells in a 100-mm culture dish at a density of 1 × 10⁶ cells in complete medium (RPMI-1640,+10%FBS).At 60–80% confluency, perform siRNA transfection using [Lipofectamine 3000]. Replace the medium 6 h post-transfection and incubate for 48 h under standard conditions (37°C, 5% CO₂). Harvest cells at 48 h post-transfection for downstream analysis.

### Western blot analysis

The cells were lysed and proteins were extracted through standard protocols. The proteins were separated by SDS-polyacrylamide gel electrophoresis and subjected to western blot analyses. Protein bands were detected by the chemi-luminescence method. Specific primary antibodies against SERPINE2 (Proteintech) was used. GAPDH (Cell Signaling) was used as a loading control.

### RNA isolation, reverse transcription and real-time PCR

Total RNA from tissues and cultured cell lines was isolated using the Trizol reagent (Invitrogen) according to the manufacturer’s instruction. Primers for real-time RT-PCR were designed using Primer Express v2.0 software (Applied BioSystems). Sequences of the primers are summarized in Additional file 1: **Supplementary Table S2**. RT was carried out with the SuperScript First-Strand Synthesis System for RT-PCR (Invitrogen) according to the manufacturer’s protocol. Real-time PCR was carried out using SYBR Green I (Applied BioSystems). The data were normalized to the geometric mean of housekeeping gene GAPDH and calculated as 2−ΔΔCT.

### Statistical analysis

The expression difference of the SERPINE2 in COAD was analyzed using the t test. The R software and Graphpad prism 9.0 software were used for statistical analysis of the data obtained from each database, and the results were visualized. Kaplan–Meier curve and log-rank test were used to analyze whether the transcription level of the SERPINEs was significantly correlated with overall survival. For statistical correlation, Spearman correlation coefficient was used according to requirements, with P < 0.05 as the threshold of significance.

## Result

**Figure 1:**
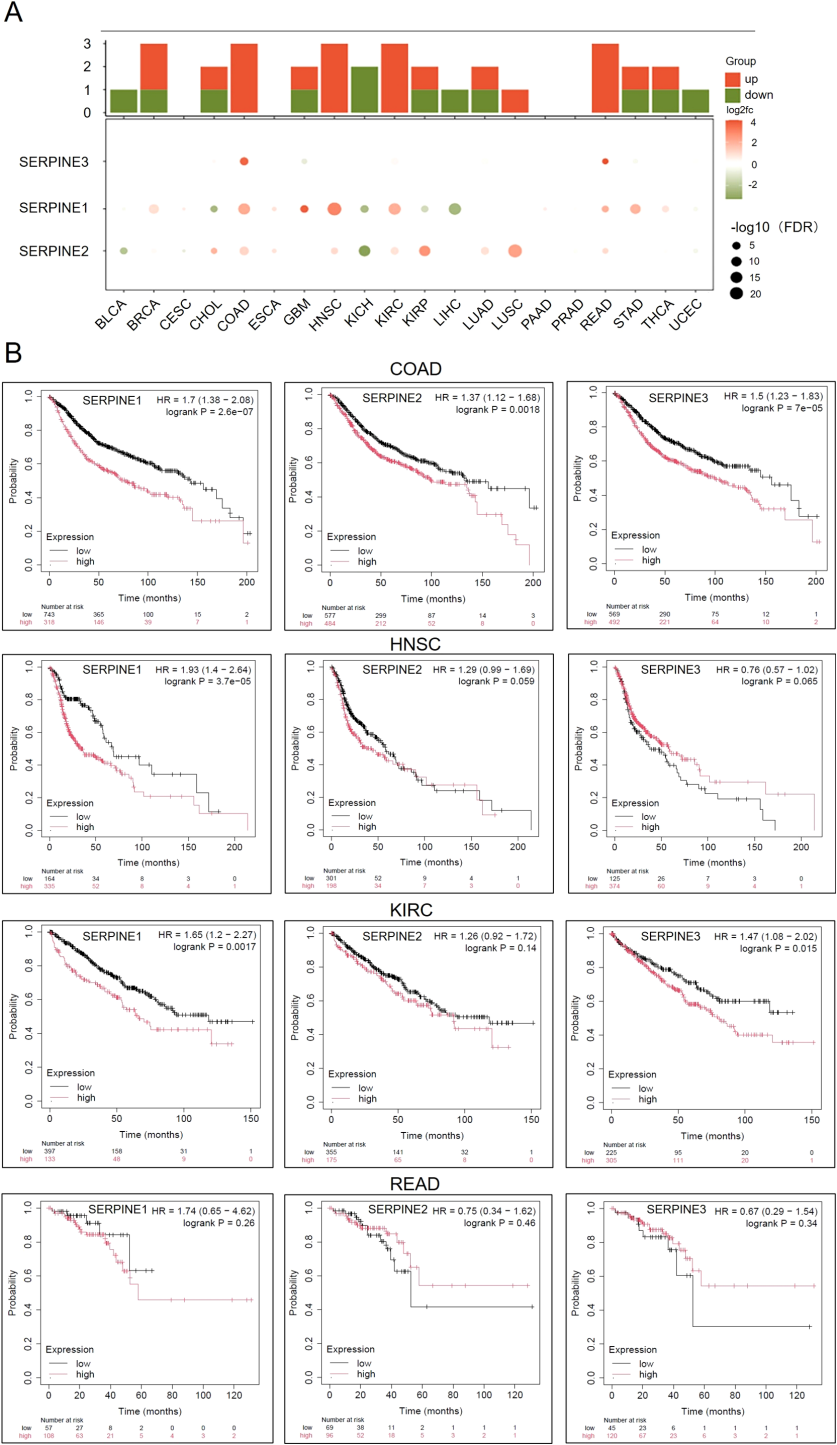
The expression levels of SERPINE family genes are upregulated in COAD. **(A)** Heatmap of mRNA expression levels of the SERPINE family genes in different tumors. **(B)** Expression of SERPINE family genes were assessed using Kaplan-Meier survival analysis in patients with COAD/HNSC/KIRC/READ.

The members of the Serine protease inhibitor E (SERPINE) family are involved in tumor growth, proliferation, and metastasis. The family includes SERPINE1-3, but the prognostic role of SERPINEs in cancer and their clinical therapeutic potential have not been thoroughly explored. Our results indicate that in the SERPINE gene family, SERPINE1 is highly expressed in pan cancer cells, followed by moderate expression of SERPINE2, while SERPINE3 shows low expression. Among Colon Adenocarcinoma(COAD)/Head and Neck Squamous Cell Carcinoma(HNSC)/Kidney Renal Papillary Cell Carcinoma(KIRC)/Rectum Adenocarcinoma(READ), the expression of SERPINE family genes was obviously upregulated (**Figure 1A**). Next, we utilized Kaplan-Meier Plotter database to confirm the prognostic value of the SERPINE family genes in COAD/HNSC/KIRC/READ (**Figure 1B**). We found that SERPINE1 posed a high-risk factor in COAD/KIRC/HNSC. Additionally, SERPINE2 played a high-risk role in COAD. However, the prognosis for the other three cancers was not significant. And we observed that SERPINE3 acted as a dangerous factor in COAD and KIRC. Survival curves illustrated that the expression of SERPINE family genes was associated with prognosis in COAD.

**Figure 2:**
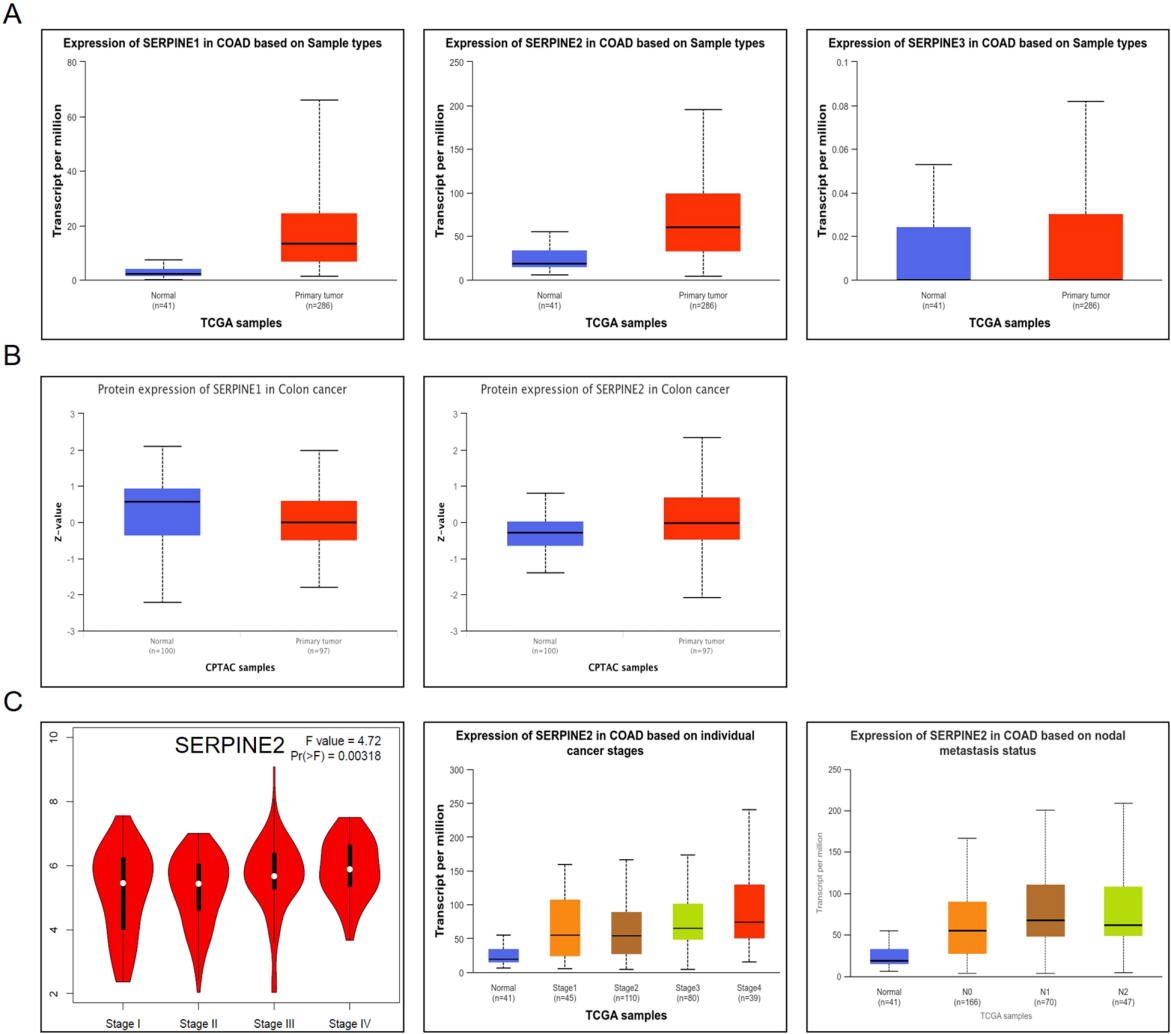
SERPINE2 expression is significantly upregulated in COAD. **(A)** Box plot of SERPINE family genes mRNA expression.in COAD(p < 0.05). **(B)** Box plot of SERPINE1 and SERPINE2 protein expression in COAD(p < 0.05). **(C)** Box plot of SERPINE2 mRNA expression among groups of different tumor grades in COAD (p < 0.05).

We utilized the ULCAN database to estimate the differential expression of SERPINE family genes in COAD. To assess differences in SERPINEs at the translational level, we compared protein levels of SERPINEs between normal and tumor groups using the CPTAC database on the UALCAN website. It is evident that the level of SERPINE1 protein is low and inconsistent with the expression of SERPINE1 mRNA in COAD (**Figure 2A**). The results showed that compared with normal tissues, the expression of SERPINE2 protein was significantly upregulated in COAD tumor tissues, consistent with its high mRNA levels. (**Figure 2B**). In addition, SERPINE3 expression was lower. Hence, SERPINE2 was selected for furth investigation in our studies. Furthermore, GEPIA and ULCAN databases revealed a close association between high expression of SERPINE2 and clinical progression in COAD patients, indicating a correlation between increased levels of SERPINE2 and malignant progression as well as lymph node metastasis(**Figure 2C**). These results suggest that transcriptional levels of SERPINE2 are significantly up-regulated in COAD patients compared to other members among the SERPINE family, making it a more promising research target.

**Figure 3:**
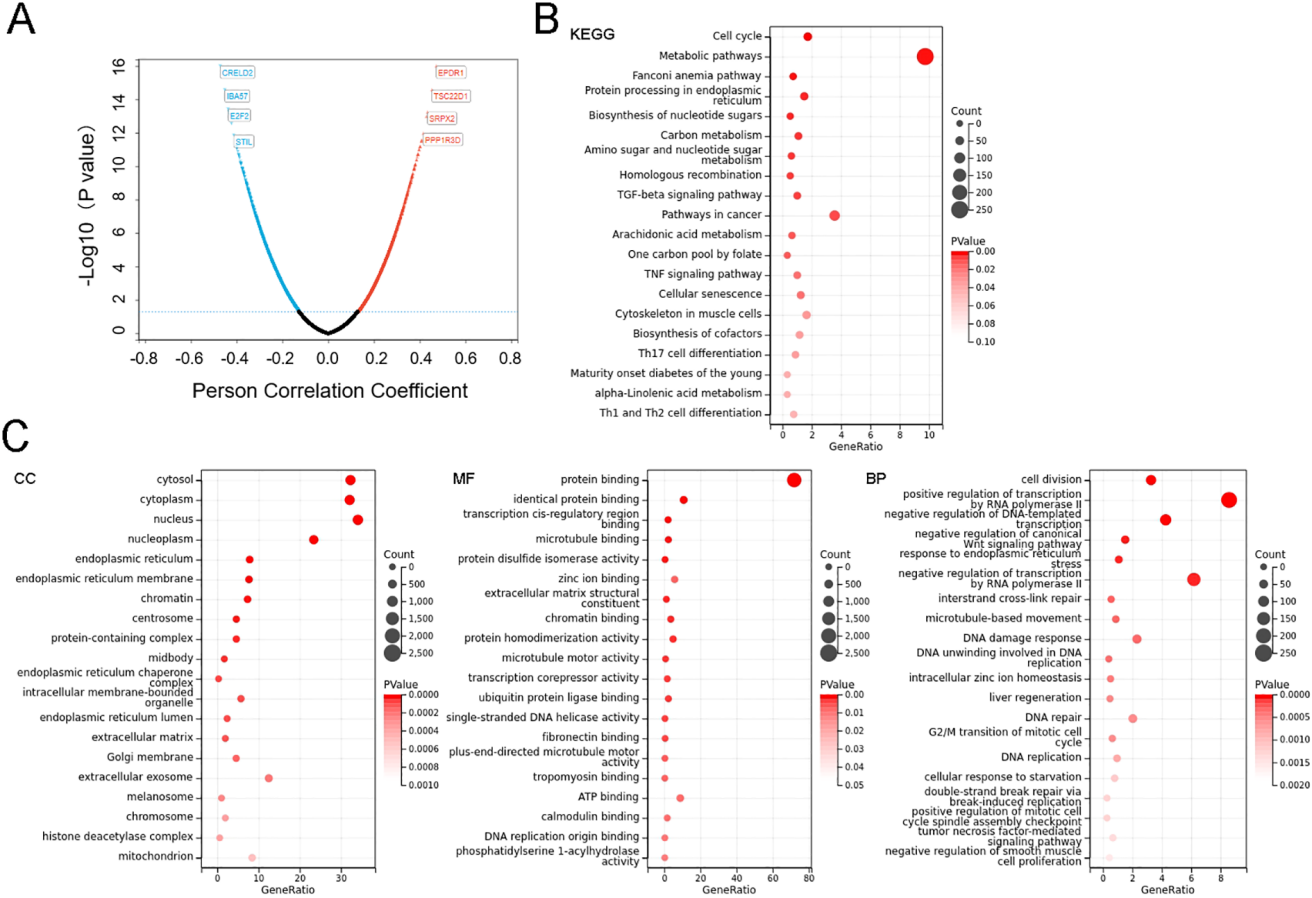
SERPINE2 expression correlates with tumor immunoregulation, division and proliferation. **(A)** Volcano map of SERPINE2 co-expressed genes in COAD. **(B, C)** GO and KEGG enrichment analysis of SERPINE2 co-expressed genes obtained from the cBioPortal, the terms p and q < 0.05 were considered to be significantly enriched. Only 20 leading gene sets are displayed in the plot.

In order to analyze the potential functions and pathways of the SERPINE2 in colorectal cancer, cbioportal database was utilized to conduct co-expression analysis of the SERPINE2 gene (**Figure 3A**). We collected all genes that were correlated with the SERPINE2 gene (|r|>0.2, p<0.05) for KEGG and GO analysis.

Based on GO analysis from the DAVID database, typical enriched GO items including biological process (BP), cellular components (CC), and molecular functions (MF) were shown in **Figure 3B**. GO enrichment analysis demonstrated that SERPINE2 co-expressed genes were primarily associated with cell division and proliferation-related pathways. Biological process (BP) annotation indicated their involvement in key cellular activities including cell division, DNA replication and cell proliferation. At the cellular component (CC) level, these genes showed significant enrichment in “chromatin”, “protein-containing complex”, and “extracellular matrix”. Molecular function (MF) analysis further identified their critical roles in molecular interactions, particularly protein binding, zinc ion binding and chromatin binding. Then, KEGG pathway enrichment was performed to explore the biological processes related to SERPINE2 expression network in COAD. The enrichment analysis of KEGG showed that the enriched pathways included metabolic pathway indicating tumor hypermetabolism, and TGF-beta signaling pathway (**Figure 3C**). The TGF-βsignaling pathway serves as a central hub in tumor immunoregulation, shaping an immunosuppressive microenvironment through multifaceted mechanisms, and is closely associated with M2 macrophage polarization.

**Figure 4:**
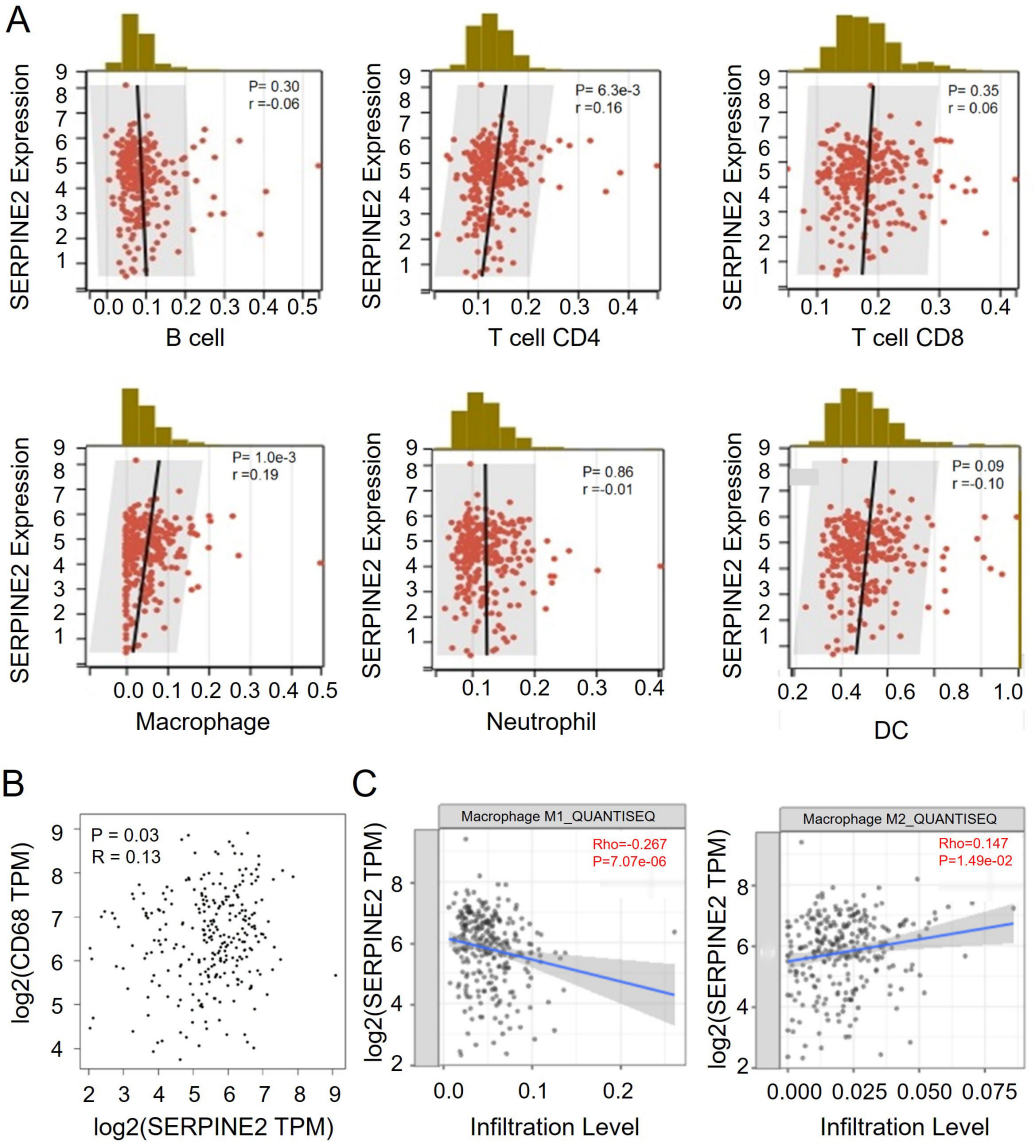
SERPINE2 is positively correlated with M2 macrophage infiltration in COAD. **(A)** Scatter plot showing the correlation of 6 different immune cell types and SERPINE2 expression (p < 0.05). The black line in each plot is a fitted linear model indicating the proportion of tropism of the immune cell along with SERPINE2 expression, and the Pearson coefficient was used for the correlation test. **(B)** Scatter plot showing the correlation of CD68+ Marcophage and SERPINE2 expression (p < 0.05). C.Scatter plot showing the correlation of M1/M2 macrophage and SERPINE2 expression (p < 0.05). The blue line in each plot is a fitted linear model indicating the proportion of tropism of the immune cell along with SERPINE2 expression, and the Pearson coefficient was used for the correlation test. (p < 0.05).

As a secreted protein, we intended to explore the impact of SERPINE2 on the tumor immune microenvironment. Through exploration in the TIMER2.0 database, we found that the CD4+ T cells and Macrophages are positively correlated with the expression levels of SERPINE2, while the infiltration of other immune-related cells is irrelevant. The correlation between macrophage infiltration and SERPINE2 expression is more significant (P=0.001, r=0.19) (**Figure 4A**). We also used another database GEPIA to analyze the correlation between SERPINE2 and macrophage marker CD68, and found that there is also a correlation between them (P=0.03, r=0.13) (**Figure 4B**). Further exploration revealed that SERPINE2 is positively correlated with M2 macrophage subtype infiltration (P=0.0149, r=0.147), while it is negatively correlated with M1 macrophage subtype infiltration (P=7.07e-6, r=-0.267) (**Figure 4C**). The results suggest that SERPINE2 may be associated with macrophage infiltration and positively correlated with M2-type macrophage infiltration.

**Figure 5:**
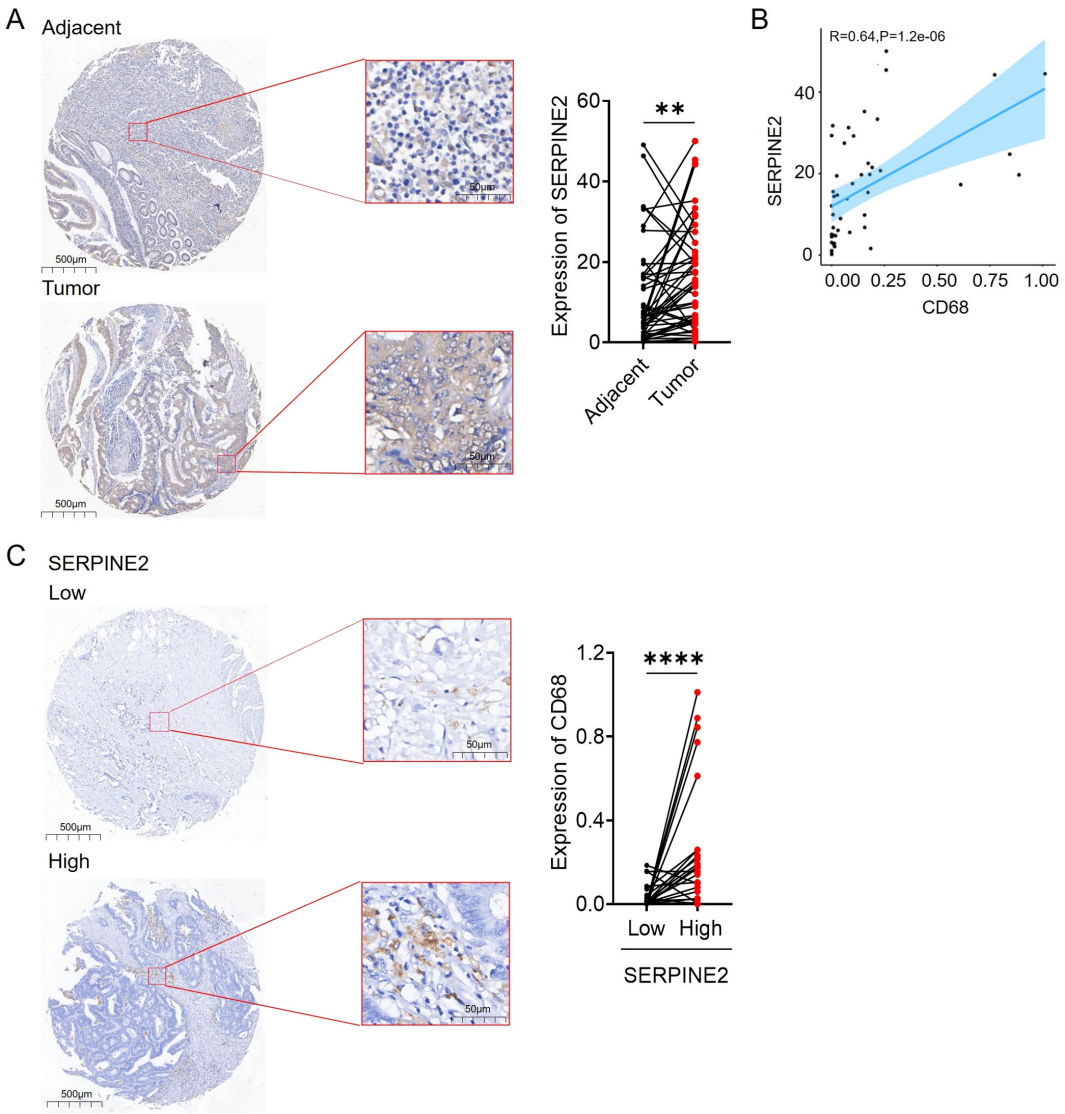
SERPINE2 is associated with infiltration of CD68 macrophage in COAD patients. **(A)** The expression of SEPRINE2 in patient tissues was detected by IHC. Adjacent tissue not expressing SERPINE2; tumor tissue strongly expressing SERPINE2. **(B)** Scatterplot showing the correlation between SERPINE2 and CD68 expression (p < 0.05). The blue line in each plot is a fitted linear model indicating the relationship between SERPINE2 and CD68 expression. **(C)** The expression of SERPINE2 and CD68 in patient tissues were detected by IHC. High levels of SERPINE2 expression are associated with increased expression of CD68. (* p<0.05, **p<0.01, *** p<0.001).

To investigate the relationship between the expression of SERPINE2 and macrophage infiltration, tissue microarray was utilized to validate the expression of SERPINE2 and CD68. The results displayed that SERPINE2 was highly expressed in cancer tissues compared to non-neoplastic adjacent tissues (**Figure 5A**). A strong correlation between SERPINE2 and CD68 was observed (R=0.64, P=1.2e-06) (**Figure 5B**). Furthermore, the expression level of CD68 was significantly increased in the cancer tissues with high expression of SERPINE2 (**Figure 5C**), suggesting that the high expression of SERPINE2 in colon cancer may promote macrophage infiltration.

**Figure 6:**
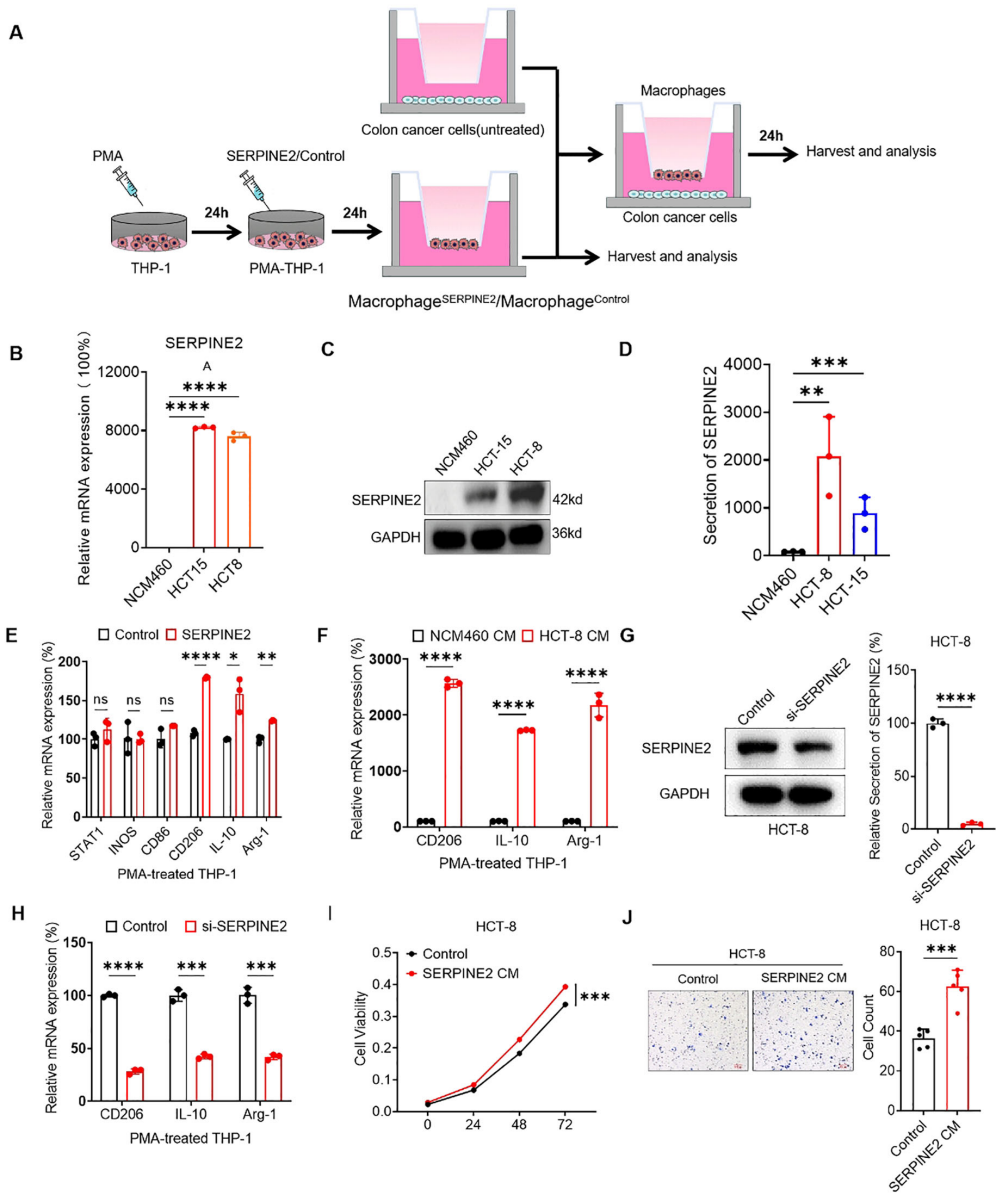
The external secretion of SERPINE2 from colorectal cancer cells promotes macrophage polarization to accelerate the development of colorectal cancer. **(A)** Schema diagram showing that THP-1 cells were treated with PMA, followed by the treatment of SERPINE2 recombinant protein as indicated treatments to obtain activated macrophages. Then tumor cells cocultured with these macrophages. were used to subsequent experiments. **(B, C)** RT-qPCR and Westen blot displayed SERPINE2 mRNA and Protein expression were upregulated in colorectal cancer cells. **(D)** The secretion of SERPINE2 in colorectal cancer cells was detected by ELISA. **(E)** The effect of SERPINE2 recombinant protein on macrophage polarization. **(F)** RT-qPCR displayed conditioned medium (CM) derived from colorectal cancer cells promotes the upregulation of macrophage M2 markers, compared to normal Intestinal cells. **(G)** ELISA analysis demonstrated that SERPINE2 secretion was significantly reduced after SERPINE2 knockdown. **(H)** RT-qPCR displayed conditioned medium with SERPINE2 knockdown down-regulated macrophage M2 markers. **(I, J)** The conditioned medium from macrophages treated with SERPINE2 recombinant protein promoted cancer cell proliferation (measured by CCK-8 assay) and migration (assessed by Transwell assay. (*p<0.05, **p<0.01, ***p<0.001).

To further elucidate the effects of SERPINE2 on TAMs, we induced THP-1 monocytic cells to differentiate macrophages by stimulation with PMA for 24 hours. These THP-1-derived macrophages (PMA-THP-1) were further stimulated with SERPINE2 recombinant protein to obtain activated macrophages (SERPINE2) (**Figure 6A**).The results showed that the mRNA level of SERPINE2 was upregulated in colon cancer cell lines compared to normal colon cell NCM460 (**Figure 6B**). Subsequently, western blot results revealed a significant increase in SERPINE2 protein expression in HCT-8 and HCT-15 compared to NCM460 (**Figure 6C**). Furthermore, the ELISA analysis of cell culture supernatants also showed a significant increase in the secretion of SERPINE2 by HCT-8 and HCT-15. Notably, the upregulation of HCT-8 is most significant (**Figure 6D**). Additionally, we found that the recombinant SERPINE2 protein may induce M2 polarization of macrophages (**Figure 6E**). When incubated with conditioned medium (CM) from NCM460 and HCT-8, it was observed that the expression of M2 macrophage markers significantly increased (**Figure 6F**). We knockdown the expression of SERPINE2 in HCT-8, collected the conditioned medium for incubation with macrophages, and observed a decrease in the polarization degree of M2 macrophage markers (**Figure 6G**). In addition, co-culture with macrophages induced by SERPINE2 recombinant protein may promote the proliferation and migration of cancer cells. (**Figures 6H-J**).

## Discussion

In view of the important roles of SERPINE gene family in progression of various tumors, it is vital to research the expression patterns and prognostic values as well as immunological roles in human cancers, which could contribute to cancer diagnosis and treatment. In this study, we conducted a comprehensive analysis of SERPINE family gene expression patterns across 33 cancer types, comparing peri-cancerous and normal tissues using TCGA-derived RNA-seq data (FPKM format) from the UCSC Xena TCGA TARGET GTEx cohort (*N* = 19,131). Following exclusion of tumors with incomplete gene records and normalization procedures, our findings were ultimately reported for 20 malignancies. similar to approaches in other article (11), We observed a significant upregulation of SERPINE expression in COAD/HNSC/KIRC/READ. Prognostic analysis confirmed the importance of SERPINEs as diagnostic markers in colorectal cancer, which was consistent in different databases and most previous studies (11). Subsequently, we conducted an analysis of various online databases and found that SERPINE2 is closely associated with malignant progression in colorectal cancer.

Serpin family E member 2 (SERPINE2), also known as PN-1, has anti-serine protease activity against thrombin, urokinase, and plasminogen (22). Previous studies have demonstrated the potential role of SERPINE2 in tumor metastasis. The extracellular serine protease inhibitor SERPINE2 fosters the metastasis of breast cancer cells by remodeling the extracellular matrix (23). Moreover, the secreted protein SERPINE2 not only drives the formation of extravascular networks but also ensures cancer cell perfusion by acting as an anticoagulant, finally leading to distant metastasis of breast cancer (24). The current explorations of SERPINE2 are insufficient to fully illustrate the diversity of its involvement in tumor development.

To dissect the gene network of SERPINE2 in colorectal cancer, we obtained a set of genes related to SERPINE2 expression in colorectal cancer through cbioportal. KEGG analysis revealed a significant enrichment of the gene set in metabolic pathways, suggesting a potential association between SERPINE2 and metabolic reprogramming in colorectal cancer. Furthermore, the TGF-β signaling pathway and TNF signaling pathway were also observed in significant enrichment. The connection between TGF-β and tumor immune-suppressive microenvironment metabolic reprogramming is crucial (25). Additionally, the TNF signaling pathway plays a vital role as an inflammatory factor that promotes polarization and maintenance of tumor-associated macrophages.

GO analysis suggests that the genes involved in co-expression mainly participate in protein interaction and cell division functions, indicating the potential existence of a large number of direct interactions and feedback loops that may impact the proliferation of cancer cells. Additionally, previous studies have indicated that SERPINE2, as an extracellular secretory factor, can bind to EGFR receptors and influence the malignant transformation of tumor cells (26). Furthermore, SERPINE2 can be internalized by fibroblasts and activate ERK and β-catenin pathways to promote cardiac cell fibrosis (27). Therefore, it is speculated that SERPINE2 may serve as an important secretory factor promoting the malignant progression of colorectal cancer.

During tumor progression and metastasis, complex communication networks between tumor cells and stromal cells determine the effect of clinical intervention (28). TAMs are the most prominent stromal cells that inhibit the anti-tumor immune response (29). Previous studies have suggested that tumors secrete specific proteins to interact with macrophages, thereby regulating the progression of the tumor. However, the mechanism of tumor-associated macrophage transformation in colorectal cancer remains unclear.

As an important secretory factor in the tumor microenvironment, we investigate the relationship between SERPINE2 and immune cells in colorectal cancer. The results indicate a close association between SERPINE2 and the infiltration of CD4+T cells and macrophages, with a more significant correlation observed with macrophage infiltration. Previous Study has indicated that SERPINE2 can bind to the CD87 urokinase receptor and induce leukocyte adhesion (30). Interestingly, we found that SERPINE2 is correlated with the infiltration of macrophage subgroups, showing a negative correlation with M1 macrophage and a positive correlation with M2 macrophage.

To further validate our hypothesis, tissue chips were used to confirm the high expression of SERPINE2 in colorectal cancer. Additionally, we also found a correlation between the expression of SERPINE2 and the macrophage marker CD68 (r= R=0.64, P=1.2e-06). Furthermore, a higher degree of macrophage infiltration was observed in the group with high expression of SERPINE2. This indicates a potential link between SERPINE2 and the infiltration of macrophages in the immune system. Current research indicates a high expression of SERPINE2 in many cancers (31). However, we aimed to validate the overexpression of SERPINE2 in colorectal cancer and explore its correlation with macrophages.

We selected the markers of M1 and M2 polarization as reported in the literature (32). We attempted to investigate whether SERPINE2 interacts with macrophages. Surprisingly, when we added recombinant SERPINE2 protein to PMA-induced THP-1 cells, we found no significant changes in M1 markers (STAT1, INOS, CD86), while there was a significant upregulation of M2 markers (CD206, IL-10, ARG-1). This suggests that SERPINE2 may induce M2 polarization in macrophages. Numerous studies have shown that tumor-associated macrophages (TAM) exhibit M2 polarization characteristics. M2 macrophages, activated by IL-4 or M-CSF, produce arginase-1 (Arg-1) (33). Additionally, they generate anti-inflammatory responses by secreting cytokines such as IL-10, participate in the generation of tumor blood vessels and remodel the extracellular matrix. Furthermore, they can control inflammatory reactions by downregulating the functions mediated by M1 macrophages and adaptive immunity, thereby playing a role in promoting tumor progression (34).

We selected normal intestinal cell line NCM460 and colorectal cancer cell lines, including HCT-8, HCT-15 to validate the expression of SERPINE2. The results indicate that the HCT-8 cell line is the most appropriate cellular model for research purpose. Not only at the mRNA and protein levels, but also the extracellular secretion of SERPINE2 shows a significantly upregulated expression in colon cancer cells.

Furthermore, we separately chose NCM460 and HCT-8 to co-culture with macrophages, thereby observing the polarization of TAMs. The M2 polarization marker showed a significant upregulation. Relevant studies have indicated that the co-cultivation of cancer cells and macrophages not only stimulates the polarization of macrophages towards the M2 phenotype, but also affects the translocation of their ecological niche (35).

We suppressed the expression of SERPINE2 in HCT-8 and subsequently co-cultured them with macrophages. As a result, we observed a significant reduction in the M2 polarization of macrophages.

Previous studies have reported that using SERPINE2 neutralizing antibodies can remodel the tumor microenvironment to suppress breast cancer metastasis (36). However, they lack evidence to directly link the SERPINE2 and TAMs.

Mechanically, we have described that the secretion of SERPINE2 by colon cancer cells may induce M2 polarization of macrophages. Subsequently, we continued to induce THP-1 with recombinant SERPINE2 protein and then co-cultured it with colon cancer cells. This led to an increase in proliferation and migration of the colorectal cancer cells. However, the specific signaling molecules that induce the malignant progression of colon cancer remain unknown. Previous studies have reported that tumor-associated macrophages (TAMs) primarily secrete anti-inflammatory cytokines such as ARG-1, IL-10, and TGF-β to generate an anti-inflammatory response, participate in tumor angiogenesis and extracellular matrix remodeling. Additionally, TAM can also control inflammatory reactions by downregulating the function of M1 macrophages and adaptive immunity, thereby promoting tumor progression (37). Here, we speculate that the SERPINE2 derived from cancer cells may stimulate tumor-associated macrophages to secrete TGF-β, leading to malignant transformation of tumors, as indicated by our previous enrichment analysis results. However, further experiments are needed for validation.

In summary, our study reveals that SERPINE2 promotes the phenotypic transformation of TAMs, establishing a new positive feedback loop between cancer cells and TAMs. This loop is crucial for the proliferation and metastasis of colon cancer, suggesting that the signaling cascade initiated by SERPINE2 may be a potential therapeutic target.

## Conclusion

In this study, we observed a high expression of SERPINE2 in colorectal cancer and identified it as an outstanding prognostic marker associated with the clinical progression of the disease. Additionally, we have uncovered a novel mechanism that SERPINE2 functions as a signaling molecule in the tumor immune microenvironment, promoting the advancement of colorectal cancer. The upregulation of SERPINE2 in colorectal cancer has been found to increase the infiltration of tumor-associated macrophages. Furthermore, we used SERPINE2 to induce the macrophages, and found that it induced M2 polarization, thereby enhancing the proliferation and migration of cancer cells. We revealed a positive feedback loop between TAMs and tumor cells.

## Data Availability

The original contributions presented in the study are included in the article/**Supplementary Material**. Further inquiries can be directed to the corresponding author/s.
