# Supplementary material for: SerpinE2 promotes M2 polarization in macrophage to accelerate colorectal cancer progression
Source: Front Oncol. 2025 May 20;15:1585935. doi: 10.3389/fonc.2025.1585935 (PMC12130048; doi:10.3389/fonc.2025.1585935)
Supplement: Supplementary file 2 [file Table2.docx]

| **Gene** | | **Primer sequence** |
| --- | --- | --- |
| **qRT-PCR** | |  |
| GAPDH | | forward: 5’- GTCTCCTCTGACTTCAACAGCG -3’ |
|  | | reverse: 5’- ACCACCCTGTTGCTGTAGCCAA -3’ |
| ARG-1 | | forward: 5’- GGACCTGCCCTTTGCTGACATC -3’ |
|  | | reverse: 5’- TCTTCTTGACTTCTGCCACCTTGC -3’ |
| CD86 | | forward: 5’- CTTCCTGCTCTCTGCTAACTTC -3’ |
|  | | reverse: 5’- CCGCGTCTTGTCAGTTTCCA -3’ |
| CD206 | | forward: 5’- GACGTGGCTGTGGATAAATAAC -3’ |
|  | | reverse: 5’- CAGAAGACGCATGTAAAGCTAC-3’ |
| IL-10 | | forward: 5’- GCCAAGCCTTGTCTGAGATGATCC -3’  reverse: 5’- GCCTTGATGTCTGGGTCTTGGTTC -3’ |
| iNOS | | forward: 5’- GACTTTCCAAGACACACTTCAC -3’  reverse: 5’- TTCGATAGCTTGAGGTAGAAGC -3’ |
| STAT1 | | forward: 5’- ATGCTGGCACCAGAACGAATGAG -3’  reverse: 5’- TCACCACAACGGGCAGAGAGG -3’ |
| SERPINE2 | | forward: 5’- CAACGCAGTGTATTTCAAGGGTC-3’  reverse: 5’- GGTGCTGATGTGTGGGATGATG-3’ |
| **siRNA** | **Primer sequence** | |
| si-SERPINE2 | forward: 5’- CAGUGUAUUUCAAGGGUCUUU-3’ | |
|  | reverse: 5’- AAAGACCCUUGAAAUACACUG-3’ | |
